# Adult-Type Ovarian Granulosa Cell Tumour: Treatment Outcomes From a Single-Institution Experience

**DOI:** 10.7759/cureus.31045

**Published:** 2022-11-03

**Authors:** Hamed Alhusaini, Mahmoud A Elshenawy, Ahmed Badran, Ayman Elshentenawy, Ahmed Mohieldin, Ahmed Mostafa Gad, Ayman Omar, Amgad Shaheen, Tusneem Elhassan, Hussein Soudy

**Affiliations:** 1 Oncology Centre, King Faisal Specialist Hospital and Research Centre, Riyadh, SAU; 2 Clinical Oncology Department, Faculty of Medicine, Menoufia University, Shebin Elkom, EGY; 3 Clinical Oncology Department, Faculty of Medicine, Ain Shams University, Cairo, EGY; 4 Clinical Oncology Department, Faculty of Medicine, Cairo University, Cairo, EGY; 5 Faculty of Medicine, Kasr Al-Ainy Center of Clinical Oncology and Nuclear Medicine (NEMROCK) Cairo University, Cairo, EGY; 6 Clinical Oncology Department, Faculty of Medicine, Zagazig University, Zagazig, EGY; 7 Clinical Oncology and Nuclear Medicine Department, Suez Canal University, Ismailia, EGY; 8 Clinical Oncology Department, Faculty of Medicine, National Cancer Institute (NCI) Cairo University, Cairo, EGY; 9 Medical Oncology, St. George and Sutherland Hospitals, Sydney, AUS

**Keywords:** inhibin, ascites, ca 125, chemotherapy, granulosa cell tumor

## Abstract

Objectives: Ovarian granulosa cell tumour is rare. This study aims to report the clinical characteristics and long-term outcomes of adult-type ovarian granulosa cell tumour (AOGCT) at King Faisal Specialist Hospital and Research Centre (KFSH&RC) and to determine the prognostic factors affecting relapse and survival.

Methods: We retrospectively reviewed patients with AOGCT, from 1988 to 2014, who were treated at our institution. Baseline characteristics, pathological findings, and outcomes were analysed and reported.

Results: Sixty-one patients with AOGCT were identified with a median age of 49 years. Median follow-up was 5.0 years (range 2.1-8.2 years). 74% of patients were FIGO (International Federation of Gynecology and Obstetrics) stage I, whereas 7% were stage II, 5% were stage III, and unknown in 14% of the cases. The most common presenting symptoms included abdominal pain (43%) and vaginal bleeding (43%). The majority of patients (38 patients, 62%) were treated with total abdominal hysterectomy and bilateral salpingo-oophorectomy. Five (8%) patients received adjuvant chemotherapy. Sixteen patients (26%) relapsed with a median time to relapse of 5.5 years (0.7-8.1 years). Half of the recurrences (eight patients, 50%) occurred after five years of diagnosis. Five-year overall survival and disease-free survival (DFS) were 93% and 84%, respectively. Factors associated with a high risk of recurrence were the presence of ascites (p=0.000) and elevated preoperative CA 125 level (p=0.048). The overall survival was significantly influenced by the menopausal status (premenopausal 100% vs. postmenopausal 84%; p=0.02), preoperative CA 125 (normal 100% vs. elevated 64%; p=0.005), ascites (present 33% vs. absent 100%; p=0.000), and age (<55 years 100% vs. ≥ 55 years 77%; p= 0.002).

Conclusion: This study confirms a good outcome for patients with AOGCT. They require long-term follow-up as late recurrences can occur many years post definitive therapy. The presence of ascites and elevated preoperative CA 125 levels were associated with a higher risk of recurrence and poor prognosis. Outcomes appear unaffected by fertility-sparing surgery or adjuvant chemotherapy.

## Introduction

Ovarian granulosa cell tumours (OGCT) are a relatively rare subtype of ovarian sex cord-stromal tumours, which are a heterogeneous group of malignancies arising from primitive sex cord cells, according to the World Health Organization classification. It accounts for 2% to 5% of all ovarian tumours [1] and includes adult and juvenile subtypes. The adult ovarian granulosa cell tumour (AOGCT) is more common and occurs in middle-aged and older women, representing 95% of all OGCTs [2]. Most patients are diagnosed with stage I disease, with an estimated five-year survival rate of more than 90% [3-5]. Patients with AOGCT may present with an asymptomatic mass or complaints of abdominal pain, and less commonly abnormal vaginal bleeding or precocious puberty may occur due to hyper-estrogenic effects [6-9].

The mainstay of treatment in AOGCT is surgery, which includes hysterectomy, bilateral salpingo-oophorectomy, and omentectomy. Lymphadenectomy as the standard practice remains controversial due to the low incidence of lymph node metastasis at initial diagnosis [10]. Fertility-sparing surgery can be an option for premenopausal women, although its role is controversial as compared with full surgical staging which should be considered following completion of child-bearing [11,12]. Additionally, the role of adjuvant therapy is uncertain due to a paucity of robust randomized evidence due to its rarity. Approximately 20%-30% develop recurrence, and late relapses can occur even beyond five years [13,14].

There is no standard approach to the management of metastatic or recurrent disease for AOGCT. Complete surgical resection can provide long-term disease control. Radiation therapy has been shown to induce clinical response and occasional long-term remission [15-18]. Platinum-based chemotherapy such as bleomycin, etoposide, and cisplatin (BEP) is the recommended treatment and produces overall response rates of 58% to 84% [19,20].

Several clinical and histopathological features might worsen the outcome of AOGCT e.g. late tumour stages at presentation, large tumour sizes (10-15 cm), tumour rupture during surgery in stage I, tumour residual after surgery, cellular atypia, and high mitotic index (4-10 mitoses per 10 high power fields) [21-22].

The ovarian granulosa cell normally produces the inhibin glycoprotein which has a negative feedback effect on the pituitary production of follicle-stimulating hormone (FSH). Higher levels of inhibin hormone might be a marker of primary or recurrent AOGCT in postmenopausal women [23].

Due to its rarity, indolent clinical course, and limited published literature with relatively small sample sizes or insufficient follow-up, studies with larger sample sizes are needed to improve our understanding. This study aims to evaluate the clinicopathologic characteristics, treatment, and long-term survival outcomes of patients treated at King Faisal Specialist Hospital and Research Centre (KFSH&RC), Riyadh, for over 30 years and to determine the prognostic factors affecting the disease-free and overall survival.

## Materials and methods

Ethical consideration

This project was conducted in accordance with the principles of the Declaration of Helsinki (2000), Good Clinical Practice Guidelines, and the policies and guidelines of the Research Advisory Council (RAC) in KFSH&RC, and approved by the Medical Ethics Committee (RAC#2141-141). The identity of the patients studied remained anonymous since no identifying data or protected health information was recorded. All data were password secured to safeguard the confidentiality of the collected patient data. This research was approved for publication by the Office of Research Affairs (ORA) and as per the internal regulation of KFSH&RC, all authors read and approved the final manuscript. This article was previously posted on Research Square on December 15, 2019

Study design and statistical consideration

This was a retrospective study with the objective of reporting real-world, single-institution experiences of AOGCT management, evaluating the clinicopathologic characteristics, treatment, and long-term survival, and determining prognostic factors affecting the disease-free and overall survival. Patient characteristics such as age, symptoms, menopausal status, parity, presence of ascites at presentation, pre-treatment Cancer antigen 125 (CA125) level, FIGO staging at initial presentation, surgery, surgical outcome, uterine pathology, adjuvant therapy, response rate, disease progression, and survival outcome, were collected. Radiographic responses were assessed retrospectively by a radiologist according to response evaluation criteria in solid tumours (RECIST) v1.1 [24]. The response in patients with the non-measurable disease was categorized according to the decision made by the treating physician.

DFS was defined as the time between surgery and recurrence, death, or last follow-up. Overall survival was defined as the time between the diagnosis and death or the last follow-up. Patients who were lost to follow-up were censored at the date of their last visit. Median follow-up was calculated by reversing the codes for death or censoring using the Kaplan-Meier method. 

DFS and overall survival were analyzed in subgroups according to age (<55years vs ≥55years), menopausal status (pre vs post-menopausal), FIGO staging at initial presentation (stage I vs stage II or III), pre-treatment CA 125 (normal vs elevated), presence of ascites, and type of surgery (conservative fertility-sparing vs non-conservative surgery).

Statistical analysis was done using the software package JMP- SAS version 9.4 (SAS Institute Inc., Cary, NC, USA). Descriptive statistics for the continuous variables were reported as mean ± 95% CI and categorical variables were summarized as frequencies and percentages. Continuous variables were compared by student’s t-test or analysis of variance (ANOVA) as appropriate, while categorical variables were compared by chi-square test. Kaplan-Meyer method was used in survival tables and curves and the different subgroups were compared by the log-rank test. The level of statistical significance was set at p < 0.05.

Procedure and data collection

Patients were considered eligible if they had histologically confirmed AOGCT. The medical records at KFSH&RC between January 1, 1988 and December 31, 2014, were reviewed and patients were identified through the hospital tumour registry software CNExT (C/NET Solutions, Berkeley, CA). A total of 61 patients were analyzed. All pathology specimens were reviewed by expert pathologists in gynecologic oncology. The above-mentioned data have been collected in a pre-designed data collection file (DCF).

## Results

Sixty-one patients with AOGCT were identified with a median age of 49 years and a median follow-up of 5.0 years (range 2.1-8.2 years). Twenty-five patients (41%) were post-menopausal. Nine patients were nulliparous. The most common presenting symptoms included abdominal pain (43%) and vaginal bleeding (43%). Sixteen (26%) patients had secondary hyperplasia, with no endometrial carcinoma observed. Only 15 (24%) patients presented with high pre-treatment tumour marker CA 125 level, The majority were FIGO stage I (74%) at presentation and 38 patients (62%) were treated with total abdominal hysterectomy and bilateral salpingo-oophorectomy (TAH+BSO), 22 patients (36%) had fertility-sparing surgery (unilateral salpingo-oophorectomy), and 98% had optimal debulking. The mean tumour size was 11.8 cm (5-27 cm); eight patients (13.1%) had a tumour size >15cm. There were no reported cases of spontaneous pre-operative tumour rupture, however, tumours ruptured intraoperatively in three (5%) patients. Fifty-five (90%) patients had no adjuvant therapy, only five (8%) patients received adjuvant chemotherapy (four cycles of BEP chemo protocol), and one (2%) patient had adjuvant hormonal treatment (megestrol acetate) (Table 1).

**Table 1:** Patients’ Characteristics No: number; NA: not available; TAH: total abdominal hysterectomy; BSO: bilateral salpingo-oophorectomy; SO: salpingo-oophorectomy; FIGO: International Federation of Gynaecology and Obstetrics system.

		No 61 (%)
Age(years)		
	Median	49
	Range	16-77
Tumour size (cm)		
	Mean	11.8
Presenting symptoms		
	Abdominal pain	26 (43)
	Vaginal bleeding	26 (43)
	Abdominal/pelvic mass	12 (20)
	Amenorrhea	4 (7)
	Asymptomatic	6 (10)
Menopausal status		
	Pre-menopausal	31 (51)
	Post-menopausal	25 (41)
	Missing	5 (8)
Parity		
	Nulliparous	9 (15)
	Multiparous	34 (65)
	Primparous	1 (2)
	NA	17 (28)
Pretreatment CA 125		
	Normal	26 (43)
	Elevated	15 (24)
	NA	20 (33)
Ascites		
	Present	7 (11.5)
	Absent	54 (88.5)
Surgery		
	BSO+/-TAH	38 (62)
	Unilateral SO	22 (36)
	Cystectomy	1 (2)
	Lymph node dissection	2 (3)
	Omentectomy	8 (13)
Surgical outcome		
	Optimal debulking	60 (98)
	Residual disease	1 (2)
FIGO stage at presentation		
	I	45 (74)
	II	4 (7)
	III	3 (5)
	Unknown	9 (14)
Endometrial pathology		
	Normal	27(44)
	Hyperplasia	16 (26)
	Polyp	1 (2)
	NA	17 (28)
Adjuvant therapy		
	No	55 (90)
	Chemotherapy	5 (8)
	Hormonal therapy	1 (2)

**Figure 1:**
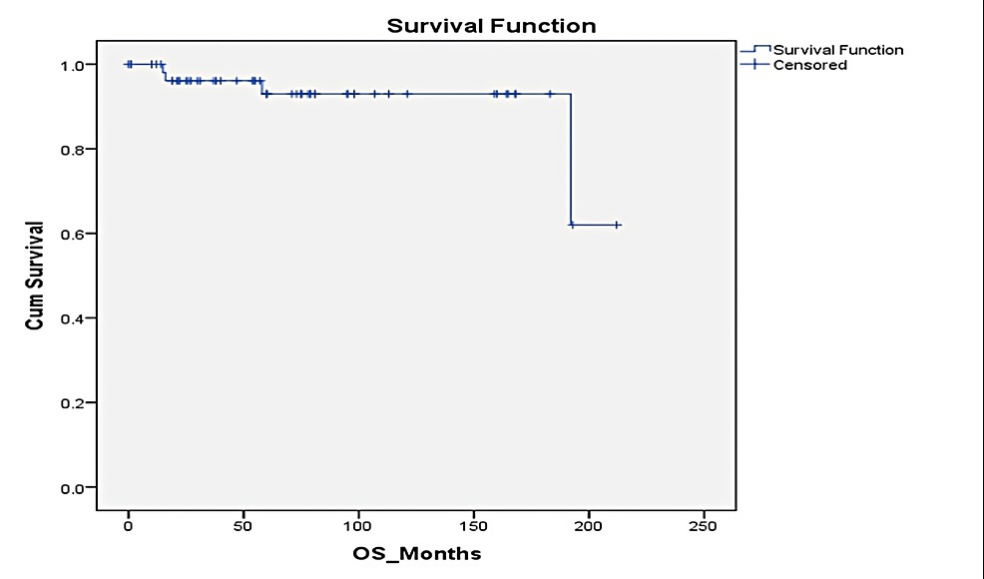
Kaplan-Meyer Overall Survival Curve

**Figure 2:**
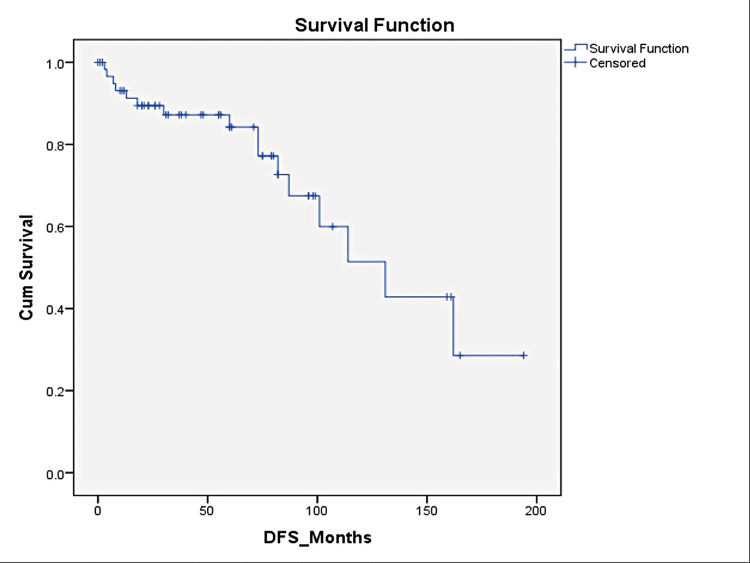
Kaplan-Meyer Disease Free Survival Curve

Sixteen patients (26%) relapsed; six (10%) had local recurrence, 10 (16.4%) developed systemic relapse, and liver involvement was the most common site of systemic relapse. The median time to relapse was 5.5 years (0.7-8.1 years). Half of the recurrences (eight patients, 50%) occurred after five years of diagnosis. Five-year overall survival and disease-free survivals were 93% and 84%, respectively (Figures 1-2).

Using univariate analysis, factors associated with a high risk of recurrence were the presence of ascites (p=0.000) and elevated preoperative tumour marker CA 125 level [>35 units/mL] (p=0.048). The overall survival was significantly influenced by the menopausal status (premenopausal 100% vs. postmenopausal 84%; p=0.02), FIGO staging at presentation (stage I 100% Vs. II&III 64%), preoperative CA 125 (normal 100% vs. elevated 64%; p=0.005), ascites (present 33% vs. absent 100%; p=0.000), and age (<55 years 100% vs. ≥ 55 years 77%; p= 0.002) (Table 2).

**Table 2:** Univariate Analysis of Different Prognostic Factors Correlated with DFS and OS DFS: Disease-free survival; OS: Overall survival; FIGO: International Federation of Gynecology and Obstetrics system.

Item		5-year DFS	p-value	5-year OS	p-value
Age					
	< 55 years	90%	0.35	100%	0.002
	≥ 55 years	70.5%		77%	
Menopausal status					
	Pre-menopausal	92%	0.98	100%	0.028
	Post-menopausal	79%		84%	
FIGO stage					
	Stage I	90%	0.14	100%	0.005
	Stages II and III	69%		64%	
Surgery					
	Fertility sparing	100%	0.063	100%	0.063
	Full surgical staging	89%		89%	
Ascites					
	Yes	0%	0.000	33%	0.000
	No	92%		100%	

The type of surgery, whether fertility-sparing versus TAH+BSO, and age (< 55, ≥ 55 years) were not significantly prognostic for both DFS and OS. Tumour rupture, lymphadenectomy, and use of adjuvant therapy were not analysed due to the relatively small number of patients included in the study. The multivariate analysis had shown ascites as the only significant factor for recurrence and lower DFS (p-value < 0.01) and the five-year OS was significantly higher in premenopausal and younger patients (<55 years) (p-value <0.001). At the date of the data collection cut-off, four (6.5%) patients died due to advanced malignancy.

## Discussion

AOGCT is a slowly progressive rare ovarian tumour, with a better prognosis when compared to epithelial tumours, though 20%-30% of them may develop late recurrence [1,5]. 

Ovarian granulosa cell tumour has different clinical behaviour and biology compared to epithelial ovarian tumours, with the sustained ability for estrogen production, which explains the prevalence of hormone dysfunction-related symptoms. In our study the main symptoms patients presented with were either vaginal bleeding (43%) or amenorrhea (7%), leading to the early diagnosis of the disease as 74% were diagnosed in stage I. This is of utmost importance, as it may lead to endometrial abnormalities with an incidence of 30%-85% endometrial hyperplasia and 3%-20% endometrial carcinoma [25,26]. In our study, hyperplasia was documented in 26% of all groups with no endometrial carcinoma found.

The mean tumour size in our report was 11.8 cm, which is matched with the average value documented in most studies, Miller and his colleagues [27], found that 33% of patients with recurrence had tumours larger than 15 cm, which were encountered in eight patients in our study. Other studies were unable to validate the prognostic significance of tumour size [28-30]. The impact of size on survival was not analyzed in this report due to the low number of patients with tumour sizes over 15 cm.

The rarity of the disease and the small number of publications are the main reasons for the absence of a prognostic model for AOGCT. However, certain common factors published in the literature had an impact on local failure rate and overall survival, among them "stage" was shown to be a prognostic factor for survival in many reports [1,5,30]. Five-year overall survival was reported to range between 75%-95% in patients with stage I disease and 22%-50% in higher stages [31-33]. Similarly, in the present study, the stage was an important factor in predicting survival; five-year overall survival was 100% in stage I disease, whereas it was 64% in stage II-III. Although not statistically significant, DFS was numerically longer in patients with stage I disease.

The prognostic value of age in AOGCT is controversial in the literature. Although Chan et al. [22] reported that age younger than 50 years was a favourable prognostic factor, Zhang et al. [28] have demonstrated that women under the age of 50 years had a 10% survival advantage in a series of 376 women, but Lee and his colleagues showed that the recurrence rate was higher at the age younger than 40 years [34]. In this study, the age of 55 years was chosen as a cut of value in analysis; survival reached 77% for the older group and 100% in the younger group, which may be an impact of menopausal status as a co-prognostic factor in this study group at this age level; this was found to be statistically significant. The five-year overall survival was 84% for postmenopausal patients.

Ascites and high tumour marker (CA125) were found to be the most important two independent factors for recurrence with an impact on survival as well. They are common presentations in epithelial ovarian tumours and are rarely reported in patients with AOGCT [35-38]. Studies on ascites and tumour marker CA125 among AOGCT are scarce. The presence of ascites is a sign of a higher stage, which was validated in literature as a strong prognostic factor for survival as mentioned above. High CA125 was reported in one study to be a poor predictor of AOGCT [39]. To our knowledge, this could be the first study to test the prognostic value of tumour marker CA125 in AOGCT, contrary to serum inhibin, which was extensively investigated [40]. This should be validated in larger studies.

Performing conservative surgical approaches includes fertility-sparing surgery which is appropriate for patients with stage I and stage II disease because of high survival and late disease recurrences [41,42]. In the present study, the performance of conservative surgery was not associated with recurrence failure and mortality, which supports its role in the fertility-sparing age group without compromising survival.

There is no prospective, randomized, controlled study evaluating the effectiveness and necessity of adjuvant radiotherapy or chemotherapy in AOGCT [43]. Therefore, the role of adjuvant therapy is controversial. In our study, 8% of the patients received adjuvant chemotherapy, 2% received adjuvant hormonal therapy and the remaining 90% of the patients were followed without adjuvant treatment and none received adjuvant radiotherapy. This is due to the low number of patients presented with stage II-III (12%), low mean tumour size, and no presurgical tumour rupture documented. In addition, optimal debulking was achieved in 98% of the cases. Despite the early stage at presentation and low-risk criteria in most of the patients, the rate of late recurrence after five years of diagnosis was high, which indicates uncovered poor risk features for future investigation, Nevertheless, 90% of the patients with recurrence underwent re-surgery.

The prognostic effect of tumour cyst rupture time, spontaneously before the operation or iatrogenic rupture during the operation is also controversial. Tumour cyst rupture was found to be associated with poor prognosis in some of the literature [34,44]. In the present study, the presence of tumour cyst rupture was noted only intraoperatively in three patients. Its effect was unable to be assessed due to low frequency.

This study has a number of limitations. This was a retrospective study. Designing prospective trials is hard in such rare diseases as AOGCT. Nevertheless, the achievement of maximal debulking in all patients in this study is one of its advantages. In addition, a relatively higher number of patients in comparison to international reports, long follow-up time (median, 97 months), involvement of patients from a single centre, performance of staging surgery including lymphadenectomy in 76.6% of the patients, and performance of surgery in 90% of the patients who had recurrence are considered points of strengths in this study.

## Conclusions

The results of our study suggest that AOGCT is often diagnosed at earlier stages, and upfront surgery with complete surgical resection should be aimed to maximize survival outcomes. The disease stage was identified as the most important prognostic factor predicting the risk of local recurrence and overall survival. Follow-up larger studies are needed to further guide optimal management for this rare but clinically important gynecologic malignancy.
